# Assessment of water, sanitation, and hygiene practice and associated factors among people living with HIV/AIDS home based care services in Gondar city, Ethiopia

**DOI:** 10.1186/1471-2458-12-1057

**Published:** 2012-12-07

**Authors:** Walelegn W Yallew, Mamo W Terefe, Thomas E Herchline, Hardeep R Sharma, Bikes D Bitew, Manay W Kifle, Desalegn M Tetemke, Mekuriaw A Tefera, Mesafint M Adane

**Affiliations:** 1 Department of Environmental and Occupational Health and Safety, Institute of Public Health, University of Gondar, Gondar, Ethiopia; 2 Boonshoft School of Medicine, Write State University, Ohio, Dayton, USA; 3 Institutes of Environmental Studies, Kurukshetra University, Kurukshetra, Haryana, India; 4 Department of Environmental and Occupational Health and Safety, University of Gondar College of Medicine and Health sciences, Institute of Public Health, P Box-196, Gondar, Ethiopia

**Keywords:** Water, Sanitation, Hygiene, Home-based care patients, People Living with HIV/AIDS, HIV/AIDS

## Abstract

**Background:**

People living with HIV/AIDS have substantially greater need for water, sanitation, and hygiene. Encouraging hygiene education for People Living with HIV/AIDS in home based care services and additional support for the provision of water, sanitation, and hygiene services is recommended.

**Methods:**

A cross-sectional study was carried during 2009 to assess water, sanitation status and hygiene practices and associated factors among People Living with HIV/AIDS in home based care services in Gondar city of Ethiopia. A systematic random sampling was used to select study subjects from 900 Home Based Care clients of People Living HIV/AIDS in Gondar city. Data was collected from 296 People Living with HIV/AIDS from two NGO’s in the city. For in-depth interview, four different categories were participated. Logistic regression and thematic framework analysis were performed for quantitative and qualitative part respectively.

**Results:**

Two hundred ninety four subjects (72.8% (214) females and 27.2% (80) males) were studied. The mean age was 35.8 ± 8.7 years. In the study, 42.9% (126) of the households have unimproved water status, 67% (197) of the households have unimproved sanitation status, and 51.7% (152) of the households have poor hygienic practice. Diarrhoea with water status; educational status and latrine availability with sanitation status; and hand washing device availability and economical reasons for the affordability of soap with hygienic practice were significantly associated. Economical reasons and hygiene education were factors that affect water, sanitation, and hygienic practice. Stigma and discrimination were minimized as a factor in the study area.

**Conclusions:**

There is high burden of water, sanitation and hygiene in people living HIV/AIDS in home based care services. Encouraging hygiene education for people living HIVAIDS in home based care services and additional support for the provision of water, sanitation, and hygiene services is recommended.

## Background

Human Immune Virus/Acquired Immune Deficiency Syndrome HIV/AIDS is one of the most devastating diseases infecting people throughout the world. At the end of 2010, the total world population affected by HIV/AIDS was estimated to be around 34 million and there were also 2.7 million new HIV infections [1]. Sub-Saharan Africa shares the largest of the global HIV burden where the rate of new HIV infections has decreased but the total number of people living with HIV continues to rise. In 2009, the number reached 22.5 million, 68% of the global. Sub-Saharan Africa has more women than men living with HIV. In 2009, there were an estimated 2.6 million people who became newly infected with HIV [2].

In Ethiopia 1.4% of adults age 15–49 are infected with HIV, with nearly 2% prevalence in women and less than 1% in men of age group (15–49) [3]. The female-to-male infection ratio of 2.1 is higher than what has been previously assumed in the Ethiopian situation. The estimated prevalence in urban areas was 10.5% (9.1% males and 11.9% females) and 1.9% in rural areas (1.7% males and 2.2% females) [3]. In Ethiopia home based care was adapted as a strategy in 2002 to support PLWHA however, the quality and duration of care are uneven largely due to stigma and lack of resources and some PLWHA are evenly abandoned [4,5].

Home Based Care (HBC) and good access to safe water and sanitation is indispensable. Water is needed for patient’s bath, washing soiled clothing and linen, and keeping the home environment clean. In addition safe drinking water is needed for taking medicines and to make food easier to eat for the patients suffering from mouth ulcers or thrush and for excellent hygiene to prevent opportunistic infections. Despite these changing needs, however, access to water, sanitation and hygiene may in fact become more difficult for households caring for PLWHA due to declining physical health, worsening economic status and/or stigma [6,7].

In Botswana home based care HIV/AIDS patients used additionally 20–80 litters of water for care from the normal water supply [8]. World Health Organization (WHO) defined access to water as “the availability of 20 litters(L)/capita/day at a distance no longer than 1,000 meters” [9]. According to the 2011 Demographic and Health Survey of Ethiopia, 54% of the population has access to an improved water source (95% and 42% for urban and rural respectively) and 82% of the population (54% and 91% of urban and rural, respectively) had no access to improved sanitation [10]. Households affected by HIV and AIDS require a greater quantity of water for bathing, washing and taking medicine, adapted sanitation facilities that meet the unique needs of the chronically ill and excellent hygiene to prevent opportunistic infections. Despite these changing needs, however, access to these services may in fact become more difficult for households caring for PLWHA due to declining physical health, worsening economic status and/or stigma [11].

HIV status had financial, health, social, and educational impacts as most PLWHA and their family members were unable to pay for their medical care [11-13]. Water and sanitation to people affected and living with HIV/AIDS were some of the shortcomings in South Africa [14]. In India time, economic constraints, lack of individual household toilets, lack of fuel for boiling water, and water scarcity were the problem for PLWHA [15]. Stigma and discrimination against PLWHAs is a significant factor affecting access to water supply [12]. Some of the factors that could motivate people to adopt safe hygienic practices, are availability of regular water supply and related sanitation facilities, encouraging stakeholder participation, introducing proper sanitation technologies and improvement of consumer sanitation knowledge [16]. Water and sanitation needs of PLWHA, access to these services often decline because of lost income, physical disability, and/or stigma associated with the disease [8,16,17]. In India, PLWHA water used for personal bathing, washing clothing and utensils increased from about 30% to more than 50% of total water consumption. Volume of water consumption for potable and non-potable purposes increased from 40 to 100 litters/ day. Bathing using soap increased from less than once a week to as often as every day [18]. In Addis Ababa, a city where the poor in general have inadequate water and sanitation facilities, PLWHA often have even more limited access than others do, due to discrimination and sickness [19]. In Gondar city HBC services are given by the two Non-governmental Organizations (NGO’s) for 900 patients. There are factors that affects individuals with PLWHA for Water Sanitation and Hygiene practice in different literatures, therefore, the present study was carried out to assess the water, sanitation and hygiene status of PLWHA and the possible factors associated with water, sanitation and hygiene in Gondar City.

## Methods

### Background

Gondar city, which is located about 750 kilometres northwest from the national capital, Addis Ababa and about 180 km from Bahir Dar city, the regional capital of the Amhara. Gondar is one of the ancient and largely populated city of the country, having a population of about 303,815 and strongly affected by HIV/AIDS epidemic having a prevalence of 10.3% [19-21]. There are two NGO which are working on home based care services for HIV/AIDS patients in all populations of the city provided services for 900 patients. Organizations for Support Services for AIDS (OSSA) provide services for 700 and Mahibere-Hiwot Ethiopia for 200 patients. Two hundred eighty and 25 voluntary caregivers were given these services for OSSA and Mahibere-Hiwot Ethiopia, respectively.

### Sampling

A cross sectional study was conducted using a pre-tested and structured questionnaire with observation which were triangulated with qualitative information obtained from in-depth interviews of patients, care givers and community members in Gondar city from April 6 –May 16, 2009. Sixty six participants from Mahibere-Hiwot Ethiopia and 230 participants from OSSA were selected by systematic random sampling. The questionnaires and checklists were first prepared in English and then translated to Amharic (native language of study participants) and again back to English to ensure its consistency. Data quality was checked every day by the principal investigator for its completeness and errors in the questionnaire. Training for data collectors and pre-test were conducted. The pre-test was conducted in Dabark city away 180 km from Gondar.

For qualitative study, 28 participants were selected and related issue were discussed. The level of saturation of information was used to decide the number of interviews. The principal investigator was moderate all the in-depth interviews. In addition, to hand written notes during the interview, interviews were tape-recorded which were later transcribed and translated into English. The main issues addressed by in-depth interview were reasons that affect the water, sanitation and hygiene status of PLWHA. Privacy and confidentiality of the clients as well as good interaction between individuals and interviewer was maintained during the data collection and interview time.

### Operational definition

Unimproved (poor) water status are water from a dam, pool, or stagnant water source from a river, stream or rainwater tank, Unprotected well, unprotected spring, water from a spring or borehole, Piped water collected more than 200 meter outside dwelling or yard) or from a water vendor. Improved (good) water status is piped water into the residence, water from household connection, Piped water collected from up to 200 m. Unimproved (poor) sanitation status is a household have no latrine or toilet facility or a bucket system; Open latrine, outside yard/compound, shared private facility, outside yard/compound, shared public facility. Improved (good) sanitation status is house hold having pour-flush latrine, Ventilated improved pit latrine, in dwelling, yard/compound. Poor hygiene practice are individuals who have no hand washing, and bathing facilities and detergents in the house, Wash their hands with water but have no soap and other detergents. Good hygiene practices are individuals have hand washing and bathing facilities with the availability of soap and other detergents in the house [22].

### Data processing and analysis

Data was checked, coded, and entered to Statistical Package for the Social Sciences (SPSS) version 15.0 statistical package for windows and analysis was made by using multiple logistic regression (Odds ratio, and 95% CI) to determine the effect of factors on the outcome variable and to control confounding effect. Analysis was made for determination of relationship between associated factors and water status, sanitation status and hygienic practices. The transcripts of the qualitative data were coded using a coding scheme and analysis was done according to selected themes.

### Ethical consideration

Ethical clearance was obtained from the Institutional Review Board of University of Gondar. Permission was obtained from the two organizations and Gondar city administration office. The questions from the questionnaire were proved not to affect the morale and personality of study subjects. Informed written consent was obtained from each study subject after explanation of, what they will take part in the research and any involvement was done after his or her complete consent. Agreement was taken, if there were risks and benefits he could be part of it. Confidentiality was ensured from all data collectors and principal investigator’s side via using code numbers than names and keeping questionnaires locked. Date collectors interview separately from other people to keep the privacy of the clients. Data collectors gave health education and advice to the clients during data collection about WASH.

## Results and discussion

### Quantitative part

#### Socio-demographic characteristics

Two hundred ninety four respondents were interviewed and the response rate was 97.9%. The mean age of respondents was 35.83 ±8.74 years. In terms of marital status 30.3% (89) were married, 27.2% (80) were widowed, 26.9% (79) were divorced and the rest were single. Amhara was the dominant ethnic group with 98% (288), and the rest was Tigre.

About 43.9% (129) of the respondents were illiterate, 13.9% (41) can read and write and remaining 42.17% (124) attended formal education from elementary up to college or university level. Regarding the occupational status of the participants, daily labourers constitute the highest one 50.3% (148) followed by merchants 10.2% (30), commercial sex workers 9.2% (27), government employees 2.4% (7), without any job 20.4% (60) and others like farmers. The income distribution reveals that the poorest/lowest, second, third/middle, fourth and richest/highest quintile accounted for 18%, 13.9%, 34%, 18.4% and 15.6%, respectively.

The number of females in present study exceeds that of males, which may be due to higher proportion of females than males, like the national incidence for single point prevalence in Ethiopia with 1.8% to males and 2.8% females [23]. About 4.1% (12), 5.6% (16), 3.4% (10), and 11.6% (34) of the clients required help during walking, eating, toilet use and cloth washing, respectively. Almost 7.8% 23) of the client had experienced diarrhoea for the past 24 hours which is comparatively lower than in Malawi and this difference might be due to the type of the container in use for water storage. Majority of the containers 90% (2580) was plastic and narrow neck and only 7% (21) used dipping for drawing water from the container while in Malawi 41.7% of container were plastic and 82.3% of respondents reported dipping a cup in the storage container of drinking water [24,25]. The slight difference might be due to hygienic and cultural practices between two countries. A similar study in Zambia and Malawi showed that PLWHA in HBC services account 27.5% and 43.3% diarrhoea for the past 24 hours [24,25].

Nationally, marital status was closely related to HIV prevalence, clients who were widowed, divorced, or separated had significantly higher rates than those who were married or living together [10]. However, in the present study the proportionally HIV prevalence was more among married 30.3% (89) than the widowed ones 27.2% (80). The middle income quintile took the largest proportion 34% (100) in the study, while the national PLWHA income quintile lie in the highest income one [10]. This may be due to the majority of the studied clients had no job and joined the OSSA and Mahibere-Hiwot Ethiopia to get support.

#### Water status of the clients

The majority of clients 71.8% (211) reported their drinking water source location as outside their yards in the neighbourhood, pipe water within their compound 5.8% (17), piped water in their own house 10.9% (32) and 9.2% (27) from public tap. Only a small number of clients i.e. 0.7% (2), 1% (30), and 0.7% (2) households get their water from protected well, spring and unprotected spring, respectively. Regarding the frequency of daily water fetching for the household of HIV clients, the majority of 44.9% (132) the households fetch water 2 times/day followed by 23.5% (69) in 3 times/day and 21.8% (64) of the household fetch once/day, and the rest 9.86% (29) households fetch their water > 4 times/day.

Assessment of size of the container for transporting water showed that majority of clients 70.7% (208) reported use of 16–20 L containers, while 5.4% (16), 10.9% (32), 6.1% (18) and 6.8% (20) of households used < 5 L, 5–10 L, 11–15 L and > 20 L containers, respectively. The 16–20 L transporting container size found in this study were similar with that in Malawi where majority of clients used 20 L containers for transporting drinking water [24]. About 96.94% of the households stored their drinking water, out of which 90.2% (258) in plastic jerricans/containers, 10.8% (31) in traditional clay pots (*Insera*), and remaining 1.7% (5) stored in metal containers. Majority of the clients 80.3% (236) practiced pouring method to withdraw water from the stored container, while 7.1% (21) practiced dipping and 12.6% (37) using both dipping and pouring methods. About 10.9% of the households treated their drinking water within 24 hours mostly through boiling 87.5% (28), chlorination (*Halazone tablets*) 6.2% (2) and filtration (candle filter) 6.2% (2). In this study showed that treat their drinking water within 24 hours. The water treatment method through boiling was also in accordance with Indian, Malawian and Zambian studies which may be due to its less cost than other treatment methods [14,24,25].

Assessment of discrimination by household members in water sources showed that 6.1% (18) of household members were discriminated in the water source use and out of them 4.4% (13) household members were forced to go far distance to fetch water to the family. In other research conducted in Ethiopia reported that one third of the respondents were discriminated in the water source and forced to go far distance to fetch water for their family [26]. The present findings were also lower from Nigerian study where 29% of respondents reported discrimination at water point. The decrease in discrimination prevalence rate in present study might be due to improvement in knowledge and attitude towards HBC by their respective family members and caregivers [11,25]. In general water status served for the clients showed that 57.1% (168) of households have improved and 42.9% (126) of the households have unimproved water status. Poor water status among clients was more likely to be one of the cause for diarrhoea among PLWHA as WHO estimates that 85 − 90% of diarrheal illnesses in developing countries can be attributed to unsafe water [26-28].

Bivarate and multivariate analysis was done between socio-demographic and predictor variables to the water status of PLWHA. The result revealed that income quintile, discrimination of family member and clients had no association in both analyzes. Whereas, educational status, forced to go far distance and need help with walking was associated in bivarate analysis and in multivariate analysis diarrhoea for the past 24 hours showed statistically significant association. Clients who have diarrhoea for the past 24 hours is 6 time more likely to be unimproved water status as compared to those who do not have diarrhoea for the past 24 hours (OR = 6.13 95% CI: 1.23, 30.57) (Table 1).

**Table 1:** Relationship between risk factors of water supply status among PLWHA on Home Based Care clients of Gondar City, 2009 (n = 294)

**Characteristics**		**General Water supply status**		**Crude OR ** **(95%CI)**	**Adjusted OR ** **(95%CI)**
		**Unimproved**	**Improved**		
Educational status	Illiterate (can’t Read and write)	58	71	1.0	1.0
	Read and write	27	14	2.36 (1.13,4.91)*	4.20 (0.92, 19.02)
	Elementary	17	34	0.61 (0.31,1.20)	0.74 (0.19, 2.78)
	Secondary	19	39	0.59 (0.31,1.14)	0.50 (0.17, 1.46)
	Above grade12	5	10	0.61 (0.19,1.89)	0.16 (0.01, 2.15)
Income quintile	Lowest (poorest)	15	38	0.47 (0.20,1.081)	0.67 (0.137, 3.36)
	Second (poor)	11	30	0.43 (0.17,1.706)	0.39 (0.057, 2.77)
	Third (middle)	52	48	1.29 (0.64,2.59)	3.55 (0.96, 13.13)
	Fourth (High)	27	27	1.19 (0.54,2.61)	2.99 (0.72, 12.42)
	Fifth (Highest)	21	25	1.0	1.0
Discrimination of family member in water source	Yes	9	9	1.35 (0.52, 3.52)	0.43 (0.06, 2.84)
	No	117	159	1.0	1.0
Discrimination of PLWHA in water source	Yes	13	22	0.76 (.36, 1.58)	1.61 (0.44, 5.86)
	No	113	146	1.0	1.0
Forced to go far distance to fetch water	Yes	9	4	3.91 (1.13, 13.47)*	3.84 (0.41, 35.27)
	No	42	73	1.0	1.0
Do you need help with walking	Yes	1	11	0.11 (0.01, 0.89)*	0.13 (0.01, 1.44)
	No	125	157	1.0	1.0
Diarrhea for the past 24 hour	Yes	15	8	2.70 (1.10, 6.59)*	6.13( 1.23, 30.57)*
	No	111	160	1.0	1.0

* Statistically significant association p < 0.05.

#### Sanitation status of the clients

More than half 57.8% (170) of the clients had latrine facility like traditional pit, VIP, pour flush latrines in 59.4% (101), 38.8% (66) and 1.8% (3) cases, respectively. About 63.5% (108) of the latrines were located inside the client’s yard, 15.8% (27) located outside the yard or shared private and 20.6% (35) of latrines are outside the yard and public shared.

Majority of 95.8% (163) the latrines did not have hand-washing facilities. Cultures of the society may affect the hand washing habit of the community, for example a Study in Myanmar showed that hand washing after toileting is influenced heavily by socio-cultural factors [29]. The clients without any type of latrine facilities 74.2% (92) opt for open defecation in the nearby fields and vacant place, 12.9% (16) reported clay pots use and the rest 12.9% (12) clients burry their faeces in their in the yard. The study demonstrated that 4.8% (14) of the clients had forced discrimination in latrine area and 5.4% (16) of the clients had forced discrimination in the latrine area which forced them to go far distance. Even if there is an improvement in discrimination, still the problem exist in the country as it was reported in similar other study in Addis Ababa, Ethiopia [19]. Another reason for not having any form of sanitation facility were economical 52.4% (65) and the rest 47.6% (59) had other reasons like lack of place, did not consider its importance and have no interest to construct. In general, sanitation status for the clients showed that 33% (97) of households have improved and 67% (197) of the households have unimproved sanitation status.

In the logistic regression analysis, sanitation status of clients is associated significantly with educational status and household latrine availability; clients who do not have latrine availability were 10 times more likely to have unimproved sanitation status as compared to those who do not have latrine availability (OR = 10.3 95% CI: 5.13, 21.03).

With increased educational status, the likelihood of having unimproved sanitation status decreased. Clients of elementary education are more than 59% less likely to be unimproved sanitation status as compared to illiterate clients (OR = 0.41 95% CI: 0.18, 0.94); Clients of secondary education are more than 71% less likely to be unimproved sanitation status as compared to illiterate clients (OR = 0.29 95% CI: 0.13, 0.63). Clients with > grade 12 are more than 74% less likely to be unimproved sanitation status as compared to illiterate clients (OR = 0.26 95% CI: 0.07, 0.94) (Table 2).

**Table 2:** Relationship between risk factors of, sanitation status among PLWHA on Home Based Care clients of Gondar City, 2009 (n = 294)

**Characteristics**		**Sanitation status**		**Crude OR ** **(95%CI)**	**Adjusted OR ** **(95%CI)**
		**Unimproved**	**Improved**		
Educational status	Illiterate (can’t Read and write)	98	31	1.0	1.0
	Read and write	32	9	1.12 (0.48, 2.61)	1.31 (0.52, 3.30)
	Elementary	29	22	0.41 (0.21, 0.82)*	0.41 (0.18, 0.94)*
	Secondary	31	27	0.36 (0.18,0 .69)*	0.29 (0.13, 0.63)*
	Above grade12	7	8	0.27 (0.09,0 .82)*	0.26 (0.07,0.94)*
Income quintile	Lowest (poorest)	32	21	0.73 (0.32, 1.68)	1.05 (0.36, 3.01)
	Second (poor)	26	15	0.83 (0.34, 2.03)	1.67 (0.56, 4.97)
	Third (middle)	68	32	1.02 (0.48, 2.16)	1.88 (0.74, 4.77)
	Fourth (High)	40	14	1.38 (0.58, 3.28)	1.73 (0.60, 4.97)
	Fifth (Highest)	31	15	1.0	1.0
Household latrine availability	Yes	86	84	1.0	1.0
	No	111	13	8.34 (4.36, 15.95)*	10.39 (5.13, 21.03)*
House hold members discriminate in the latrine	Yes	11	3	1.85 (0.50, 6.80)	4.18 (0.52, 33.28)
	No	186	94	1.0	1.0
Have you discriminated in the latrine	Yes	11	5	1.08 (0.36, 3.22)	0.30 (0.05, 1.62)
	No	186	92	1.0	1.0
Do you need help with walking	Yes	10	2	2.54 (0.54, 11.82)	1.57 (0.26, 9.49)
	No	187	95	1.0	1.0
Diarrhea for the past 24 hour	Yes	14	9	0.74 (0.31, 1.79)	0.61 (0.19, 1.87)
	No	183	88	1.0	1.0

* Statistically significant association p < 0.05.

A study in Bangladesh also investigated that educational status is highly significant to the presence of sanitation facilities i.e. household [30]. Cultures of the society may affect the hand washing habit of the community, for example a Study in Myanmar showed that hand washing after toileting is influenced heavily by socio-cultural factors [29]. This indicates that in the study area people may only consider the availability of latrine, but did not consider hand-washing facilities.

#### Hygienic practice of the clients

Only 8.2% (24) of the clients had hand washing place, from those who have a location for hand washing, 4.1% (12) households have hand washing devises like tap, basin, bucket, sink). About 58.8% (173) clients washed their hands with soap during the past 24 hours of a day. Only 21.7% and 8% clients in Malawi and Zambia, respectively using soap for washing hands after defecation, while 45% in Zambia and 58.8% (173) in the study area clients wash their hands with soap during the previous 24 hours, this difference may be due to people gave priority for other activities rather than for washing their hands after defecation [24,25]. About 39.1% (115) of the clients afford soap regularly for hygienic purpose while remaining could not. Out of those clients who did not afford soap, mostly 56.8% (167) because economical reasons but the rest have their own perceptions. In general 48.3% (142) of the households had good hygienic practice, while remaining 51.7% (152) had poor hygienic practice. Almost half of the clients 49.3% (145) had attended hygiene education in the past one year. The sources of information about water, sanitation and hygiene for the clients were, 51.0% (140) from voluntary home care givers, 43.4% (127) from health institutions and 5.5% (17) from public meetings.

The study showed that educational status is associated significantly with hygienic practice. Individuals of who can read and write are 2.4 times less likely to have poor hygienic practice than those illiterate clients (OR = 2.4 95% CI: 1.06, 5.41). This finding was similar with other researches in Myanmar and South Africa [6,29,31]. Hand washing device availability is associated significantly with hygienic practice. Clients who do not have hand washing device is 8.7 times more likely to have poor hygienic practice compared to those who have hand washing device in the house (OR = 8.76 95% CI: 1.00,76.72). Economical reasons for the affordability of soap are associated significantly with hygienic practice (OR = 0.04 95% CI: 0.00, 0.42). There was no significant association between income quintile, frequency of bathing, presence of towel, attending hygiene education and diarrhoea for the past 24 hours (Table 3).

**Table 3:** Relationship between risk factors of Hygienic Practice among PLWHA on Home Based Care clients of Gondar City, 2009 (n = 294)

**Characteristics**		**Hygienic Practice**		**Crude OR (95%CI)**	**Adjusted OR (95%CI)**
		**Poor**	**Good**		
Educational status	Illiterate (can’t Read and write)	64	65	1.0	1.0
	Read and write	27	14	1.95 (0.94, 4.07)	2.40 (1.06, 5.41)*
	Elementary	25	26	0.97 (0.51, 1.86)	1.11 (0.54, 2.28)
	Secondary	28	30	0.94 (0.51, 1.76)	1.11 (0.55, 2.22)
	Above grade12	8	7	1.16 (0.39, 3.38)	1.29 (0.41, 4.06)
Income quintile	Lowest (poorest)	26	27	1.14 (0.51, 2.53)	1.39 (0.57, 3.38)
	Second (poor)	19	22	1.02 (0.44, 2.39)	1.32 (0.52, 3.33)
	Third (middle)	54	46	1.39 (0.69, 2.81)	1.64 (0.76, 3.50)
	Fourth (High)	32	22	1.73 (0.78, 3.83)	2.39 (0.99, 5.76)
	Fifth (Highest)	21	25	1.0	1.0
Hand washing device availability	Yes	1	11	1.0	1.0
	No	151	131	12.67 (1.61, 99.52)*	8.76 (1.00, 76.72)*
How frequently do you wash your body	Every day	8	7	1	1
	Twice a week	45	44	0.89 (0.29, 2.67)	1.31 (0.38, 4.54)
	Once a week	79	78	0.88 (0.30, 2.56)	1.03 (0.31, 3.40)
	once in a month	16	10	1.40 (0.38, 5.06)	1.62 (0.36, 7.14)
	more than a month	4	3	1.16 (0.19, 7.11)	12.80 (0.81, 20.88)
The reason that soap not affordable	Economical	104	63	0.12 (.02, 0.54)*	0.04 (0.00, 0.43)*
	others	8	4	1.0	1.0
The presence of towel in the HW place	Yes	6	15	1.0	1.0
	No	146	127	2.87 (1.08, 7.62)*	2.26 (0.72, 7.09)
Attending hygiene education	Yes	73	72	0.89 (0.56, 1.42)	1.23 (0.73, 2.07)
	No	79	70	1.0	1.0
Do you need help with walking	Yes	3	9	0.29 (0.07, 1.12)	0.23 (0.04, 1.14)
	No	149	133	1.0	1.0
Diarrhea for the past 24 hour	Yes	15	8	1.83 (0.75, 4.46)	1.62 (0.61, 4.26)
	No	137	134	1.0	1.0

* Statistically significant association p < 0.05.

#### Qualitative part

Twenty eight persons participated in in-depth interview from four categories such as HBC voluntary caregivers, community members, PLWHA those in HBC services and family caregivers. The participants were residents of Gondar city having age between 21–54 years with educational level ranged from illiterate to 12-grade complete. Important guiding questions were forwarded for all participants including some questions in the quantitative part that need further probing. All the participants of the in-depth interview know how water, sanitation, and hygienic practice are important for PLWHA. The findings was presented according to selected themes.

Economical problem was the major reason mentioned by majority of the respondents for insufficient amount of water availability in the household. All respondents believed that additional water is necessary for PLWHA other than the normal individual for different purpose like “taking.

Majority of the respondents expressed their “*thanks for Anti retroviral Treatment (ART)*” that people starts considering HIV diseases as a normal diseases with in previous 2 years. Due to this most of the time stigma and discrimination become minimized in their area, however, in some areas the problem exists,

“I have two children; my husband is passed two years before. Even if there is some progress to my health, I don’t have any job, due to my HIV status our neighbours forbid me to touch their water faucet, unable to transmit the diseases to their family, now I am forced to fetch water from the spring by travelling long distance from my house.”

(*A 34 year-old married woman with two children)*

In another case explained by a female living in one small living room, after her husband died from HIV before two years explained:

“One day, In the morning after I used the latrine, the owner of the household called me and told me that, I heard that your husband is dead by AIDS, you are also positive, so we should take the preventive measure, after this time you can’t share this toilet with us, because it needs additional water and the virus may evaporate and transmit with us. After that, now I am using public latrine by payment after travelling a long distance”

(*29 years female clients with 5 children)*

As found in quantitative study, majority of the respondents responded that economical reasons were the main contributor regarding latrines unavailability in the clients households. Discrimination and sickness were also the other causes. Majority of participants knew that how sanitation facilities are important for PLWHA for their waste disposal.

Most of the depth interview results were triangulated to the descriptive result showed that majority of clients were unable to have good hygiene practices in the household was due to economical reasons. Discrimination and sickness were also the cause to poor hygienic practices for few responds, while, based on the view of majority of respondents discrimination in the hygienic practices, and in general become minimized. Studies in other areas like in South Africa, Nigeria, and Kenya showed that economical reasons are the major reasons for poor hygienic practice for PLWHA in HBC services [32-34]. In other studies in Democratic Republic of Congo, South Africa, Ethiopia and Nigeria showed that in addition to economical problems, the attitude of care givers, and their educational status were the core determinant factors towards the general hygiene status of the clients [35-38]. The result obtained in this study was likely to be due to the low-income status of the subjects, which might not have afforded them the practice of good hygiene practice in the household in addition to other factors.

All participants know that how sanitation facilities are important for PLWHA. hygienic practices like, washing body, washing hands, washing clothes and shaving/cutting their hair were some of the activities that majority of respondents expressed, patients practiced on their daily activities. Almost all respondents express soap and water are the most important equipment that are important for keeping hygienic practices. The main reasons for not using soap by the clients were also assessed, majority of the qualitative study respondents agreed that it prevents communicable diseases by removing dirt and odour from the body. Most of the respondents expressed that economical issue was the major problem for clients to keep their hygienic activities. One woman at the age of 47 expressed that, one of her neighbour forbid her to washcloth equipments (*tisht*) after knowing her HIV status, *“Your dirt may remain in the washcloth equipment and it may transmit to us thereby also transmitting the virus to us”*.

In general majority of respondents agreed that after ART majority of patients are started to work, even if some patients are still in bed ridden, stigma and discrimination in the hygienic practices became minimized, compared to the previous years, but economical problem remain the burden for most of the patients still at this moment a 25 years old women said that “*Everything concerning this illness is tied to money”.*

### Limitation of this paper

It may be difficult to generalize results to patients in other institution and localities because of differences in socio-economic, educational status and lifestyles. Lack of a baseline study in the study area for home-based care programme to compare.

## Conclusions

Almost half and two third of PLWHA in HBC services had poor water and sanitation conditions. Frequent illness (diarrhoea) is associated with poor water status of the households. Increased educational status decreased the likelihood of having poor sanitation status. Almost half of PLWHA in HBC services were found to have poor hygienic practices. Discrimination, economical reasons, hygiene education, and sickness were some of the factors that affect water, sanitation and hygiene in this study.

Water, sanitation, and hygiene problems are very common among HIV/AIDS patients in HBC services and attention should be given to water, sanitation and hygiene beside other preventive and control methods. Support to PLWHA in HBC services for the provision of water, sanitation and hygiene services. Reduce barriers to improve water, sanitation, and hygiene behaviour among PLWHA by assessing the feasibility of supporting practical, effective, and locally appropriate materials that will improve safe water, sanitation and hygienic practices. Identify sectors involved in water, sanitation and hygiene and for collaboration and strengthen HIV/AIDS program for future interventions. Enhance training for volunteers’ and HBC clients towards water, sanitation and hygiene. Health professionals working on water, sanitation and hygiene should give appropriate information for the linkage between water, sanitation and hygiene and HIV and its prevention. Water, sanitation and Hygine should be included in the package of ART for PLWHA.

## Competing interests

The authors declare that they have no competing interests.

## Authors’ contributions

WWY contributed to the data collection, study methodology, analyzed and wrote the manuscript. MWT literature review and analyzed the data , TEH literature review and analyzed the data, HRS literature review and analyzed the data, BDB Co-wrote the manuscript, MWK analysis and edit the manuscript, DMT Co-wrote the manuscript, MAT literature review and analyzed the data and MMA Literature review and editing the manuscript. All authors read and approved the final manuscript.

## Pre-publication history

The pre-publication history for this paper can be accessed here:

http://www.biomedcentral.com/1471-2458/12/1057/prepub
